# A Cantilever-Based Piezoelectric MEMS for Arbitrary XY Path Generation

**DOI:** 10.3390/mi13091514

**Published:** 2022-09-13

**Authors:** Fabio Botta, Andrea Rossi, Nicola Pio Belfiore

**Affiliations:** Department of Industrial, Electronic and Mechanical Engineering, Roma Tre University, 00146 Roma, Italy

**Keywords:** MEMS, piezoelectric, path generator

## Abstract

This work pertains to the design of a cantilever-based piezoelectric MEMS device that is capable of generating arbitrary paths of its tip. The conceived device consists of a pair of rigidly coupled piezoelectric bimorph cantilevers, and a theoretical model is developed for the analytical evaluation of the proper voltage distribution to be supplied to the inner and outer electrodes of each piezoelectric actuator, in order to drive the tip along any desired trajectory. Such a device could be appealing in some microsurgical operations, i.e., the unclogging of arteries, endoluminal treatment of obstructive lesions, but also as a 2D micropositioning stage, etc. Theoretical predictions of voltage versus time that allow several pathways such as circles, ellipses, spirals, etc., to be accomplished have been verified with multiphysics FEM simulations and the numerical outcomes seem to corroborate the proposed model.

## 1. Introduction

The actual micro-electro-mechanical systems (MEMS) fabrication capabilities fostered the deployment of new micro-devices for micro-object manipulation [1,2], micro-positioning [3], energy harvesting [4], sensors [5], Lab-on-Chip [6], etc. Nowadays, the micro- and nano-machining developments allow to realize multi-hinge and multi-DoF MEMS devices. The main issue lies in the design and fabrication of lumped flexure hinges that withstand the elastic deformations of the entire system. Novel microdevices based on Conjugate Surface Flexure Hinges (CSFHs) were conceived and fabricated [7] to improve their kinematic and mechanical reliability. A CSFH is a particular flexure consisting of a curved beam, which provides compliance, and a portion of conjugate-profiles. When the elastic weight center of the curved beam overlaps with the conjugate profile center, the parasitic deformations are minimized [8].

In kinematic synthesis, the three typical problems consist in function generator, path generator and rigid body guidance [9]. The earliest examples can be found in the late 19th century [10] and in the early 20th century [11,12]. A D-Drive MEMS device for straight line path generation based on a four-bar linkage mechanism, which can be useful in industrial and biomedical applications, has been recently proposed [13]. The Scott–Russell mechanism was recently adopted to conceive a novel parallel motion compliant gripper [14]. This mechanism was successfully exploited to implement piezoelectric micropositioning devices that provide pure translational motion of the jaws and allow to simplify the geometry of the whole system [15,16,17]. Lately, a microgripper for atherectomy operations based on Stephenson’s kinematic chain has been proposed [18]. The tip motion of the microgripper was designed to avoid perforating the lumen walls while removing obstructions. However, very scarce efforts have been made in the literature to devise microdevices capable of performing an arbitrary path or for rigid body guidance.

Unfortunately, such microsystems still show motion capability limited to single or few degrees-of-freedom (DoF), and their nature is usually restricted to translations or rotations [19]. Actuation at the micro- or nano-scale is a rather difficult task [20] and so it is quite difficult to build a microsystem that is able to control a path at the micro- or nano-scale.

Among the large variety of technical difficulties in building downscaled devices [21], the actuation of multi-DoF (Degrees of Freedom) micro- or nano-systems is rather cumbersome because it makes the control of the effects of single or multiple actuators more difficult. Possible strategies consist in resorting to control techniques [22,23], kinematic synthesis [13,24] or topology [25]. In this area, the development in recent years of smart materials can be of great help. Among them, piezoelectric materials are widely used in various fields such as vibration damping [26,27,28,29], energy harvesters [30,31], etc. The piezoelectric actuation has been increasingly adopted and offers an alternative to the more widespread electrostatic and thermal actuation systems. Actually, MEMS devices based on piezoelectric actuation can be used as optical scanners [32], resonators [33,34], sensors [35,36] and wearable devices [37]. Several researches focused on piezoelectric actuator-based microgrippers to provide micro object manipulation [38,39,40,41] or micropositioning stage [42,43,44].

In the present paper the actuation task is based on the piezoelectric effect [45]. Here, the proposed microdevice consists of two piezoelectric bimorph beams interconnected by means of a flexure hinge consisting of a C beam. The overall length of such a device is 1 mm and its free end is capable of reproducing any arbitrary pathways but only in the xy plane. The accurate motion is accomplished by supplying the electrodes of the piezoelectric layers with the proper voltage. Thereby, the cantilever tip is capable of tracing any trajectories, for instance circles, ellipses, spirals and even more intricate paths. The work provides a theoretical model which is capable of predicting the driving voltage that has to be supplied to the piezoelectric actuators for generating any trajectory. The theoretical model has been verified with multiphysics Finite Element Analysis (FEA) simulations and the results are consistent with the analytical data.

In what follows, the theoretical model of actuation of the proposed microdevice is introduced (Section 2), the FEA simulation setup and the corresponding results are presented in Section 3.

## 2. Analytical Model

**Figure 1:**
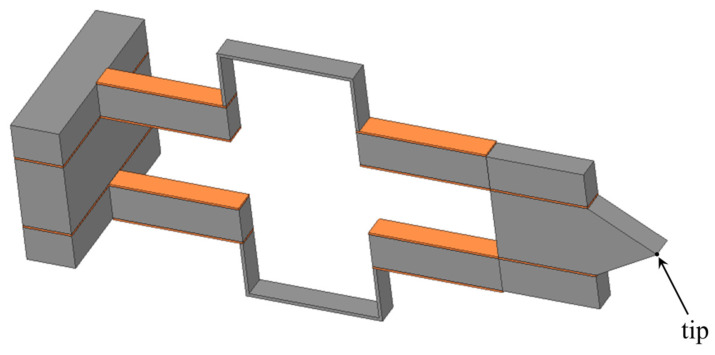
Geometry of the proposed piezoelectric-based MEMS device.

**Figure 2:**
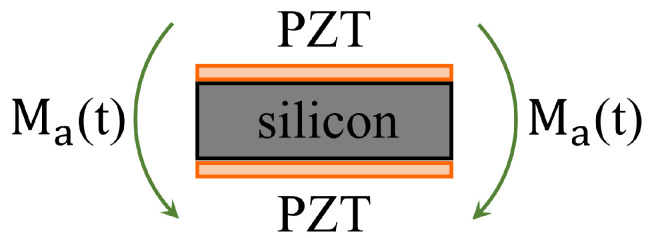
Piezoelectric actuator actions on the beam under the assumption of perfect bonding among the piezoelectric layers and the structure.

Figure 1 shows the schematic of the structure:

The point P is the point that describes the desired trajectory. It is possible to observe that the structure is symmetrical with respect to the *x*-axis and that the point P is on the same axis, so that if a x-symmetrical load is applied to the structure, the point P will only be able to have a x-symmetrical displacement. In other words, its displacement can only occur along the *x*-axis. Instead, if the load is x-antisymmetric the displacement of the point P will be x-antisymmetric, i.e. only along the *y*-axis. Piezoelectric action on the beam is shown in Figure 2. It consists of two bending moments concentrated at the end of the piezoelectric plates where (see [28,29,46]):

(1) $$M_{a}\left(t\right)=\frac{\psi }{6+\psi }E_{a}bt_{a}t_{b}\Lambda \left(t\right)$$

and

(2) $$\left\{\begin{array}{c} \psi =\frac{E_{b}t_{b}}{E_{a}t_{a}}\\ \Lambda \left(t\right)=\frac{d_{31}}{t_{a}}V\left(t\right) \end{array}\right.$$

**Figure 3:**
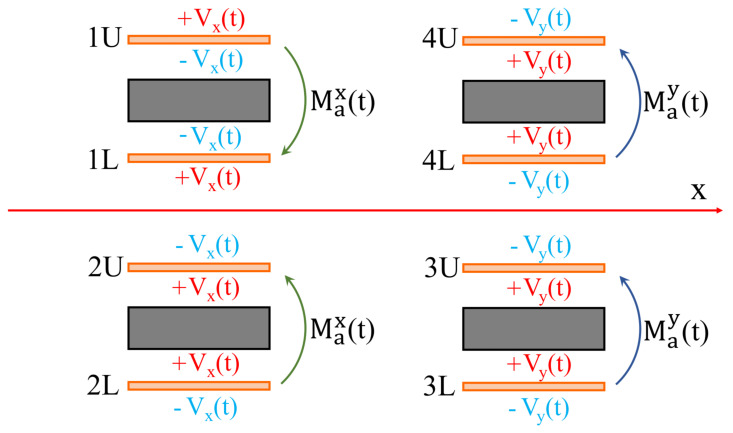
Example of symmetrical and antisymmetrical loads with respect to the *x*-axis. $M_{a}^{x}\left(t\right)$ and $M_{a}^{y}\left(t\right)$ denote respectively the bending moments which produce only *x*-axis and *y*-axis motion of the tip.

The orientation of the moments depend on how the piezoelectric plates are fed. In Figure 1 it is possible to observe that there are two sections: x-displacement and a y-displacement. In the first section the plates are fed in such a way as to apply two symmetrical moments with respect to the *x*-axis (the point P moves only along the *x*-axis), while in the y-displacement section the electrical potential distribution is such that the piezoelectric plates apply two antisymmetrical moments (the point P moves only along the *y*-axis) see Figure 3.

Since the displacement of point P is directly proportional to the applied moment M, the following can be written:

(3) s(p)=ui+vj

with:

(4) $$\left\{\begin{array}{c} u=C_{x}V_{x}\left(t\right)\\ v=C_{y}V_{y}\left(t\right) \end{array}\right.$$

where the constants $C_{x}$ and $C_{y}$ depend on geometry, material properties, boundary conditions, etc. Substituting Equations (4) into Equation (3) gives:

(5) $$s\left(p\right)=C_{x}V_{x}\left(t\right)i+C_{y}V_{y}\left(t\right)j$$

So, given the parametric equations of the desired trajectory:

(6) $$\left\{\begin{array}{c} x_{p}\left(t\right)=f_{x}\left(t\right)\\ y_{p}\left(t\right)=f_{y}\left(t\right) \end{array}\right.$$

it will be sufficient to choose the voltage functions $V_{x}\left(t\right)$, $V_{y}\left(t\right)$ similar to $x_{p}\left(t\right)$, $y_{p}\left(t\right)$:

(7) $$\left\{\begin{array}{c} V_{x}\left(t\right)=\overset{\wedge }{V_{x}}\cdot f_{x}\left(t\right)\\ V_{y}\left(t\right)=\overset{\wedge }{V_{y}}\cdot f_{y}\left(t\right) \end{array}\right.$$

The constants $\overset{\wedge }{V_{x}}$ and $\overset{\wedge }{V_{y}}$ govern the amplitude of displacement in x− and y− directions (refer to the following Section for details). Thus, it is possible to achieve any trajectory.

## 3. Results and Discussion

**Figure 4:**
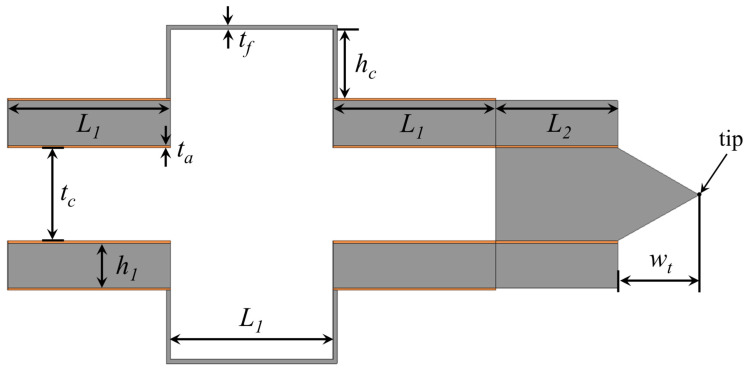
Relevant dimensions of the proposed piezoelectric-based MEMS device.

The proposed microdevice has been verified by a multiphysics FEM software (COMSOL). The details of the geometry are reported in Figure 4 while the geometric values of the different quantities are reported in Table 1.

Linear isotropic elasticity was considered for the structural material (silicon) while the piezoelectric actuators (PZT-5A) were modelled according to the strain-charge formulation (see Table 2). The geometry of the conceived piezoelectric microsystem allows to exploit the plane stress and strain approximation, so that the 2D geometry can be considered and the computational efforts decrease.

As a proof of the microdevice’s ability to make the tip trace arbitrary trajectories, many trajectories relevant in the field of micro-invasive surgery as well as surface scanning were considered. Circular, elliptical, spiral and cycloidal trajectories are particularly suitable in atherectomy operations to remove obstructions and calcifications developing within arterial walls [18,47] so long as they entail a low risk of perforating the lumen walls. Lissajous and cosinusoidal/linear trajectories are used as alternative scanning methods in atomic force microscopes (AFM) [48]. Such pathways allow the sample’s surface to be scanned more rapidly than the consolidated raster method. Appropriate electric potential functions $V_{x}\left(t\right)$ and $V_{y}\left(t\right)$ must be delivered to the piezoelectric actuators of the x and y displacement units (see Figure 1) in order to reproduce such trajectories. For each trajectory under investigation, the functions $V_{x}\left(t\right)$ and $V_{y}\left(t\right)$ can be found in Table 3.

**Figure 5:**
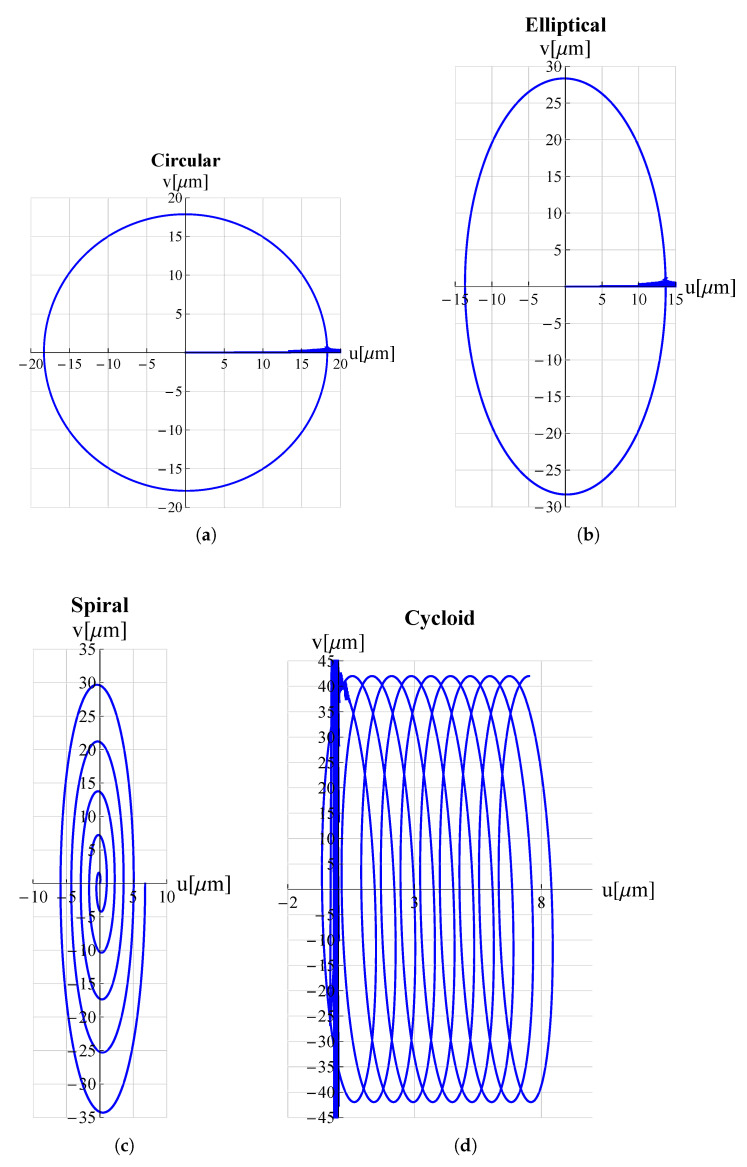
Trajectories, travelled by the proposed microdevice’s tip, applicable for atherectomy operations: (**a**) circular, (**b**) elliptical, (**c**) spiral and (**d**) cycloidal. *u* and *v* are displacement components along *x*-axis and *y*-axis respectively. All graphs were obtained by means of FEM simulations.

**Figure 6:**
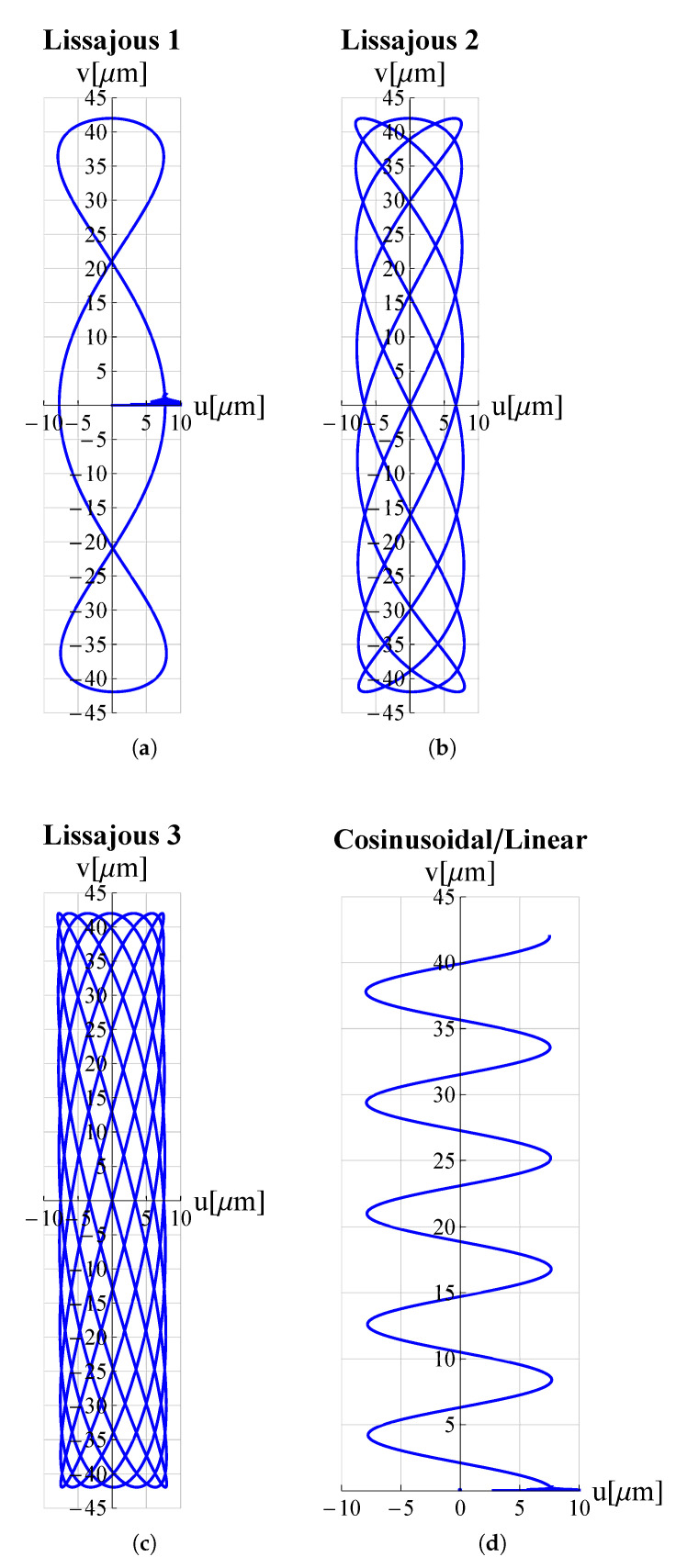
Trajectories, travelled by the proposed microdevice’s tip, applicable for scanning methods in atomic force microscopes (AFM): (**a**) Lissajous 1, (**b**) Lissajous 2, (**c**) Lissajous 3 and (**d**) cosinusoidal/linear. *u* and *v* are displacement components along *x*-axis and *y*-axis respectively. All graphs were obtained by means of FEM simulations.

The results of the FEM simulations are reported in Figure 5 and Figure 6. It can be seen that the results perfectly trace the desired curves. However, this amplitude can be adjusted, according to different applications, by acting on $\overset{\wedge }{V_{x}}$, $\overset{\wedge }{V_{y}}$.

**Figure 7:**
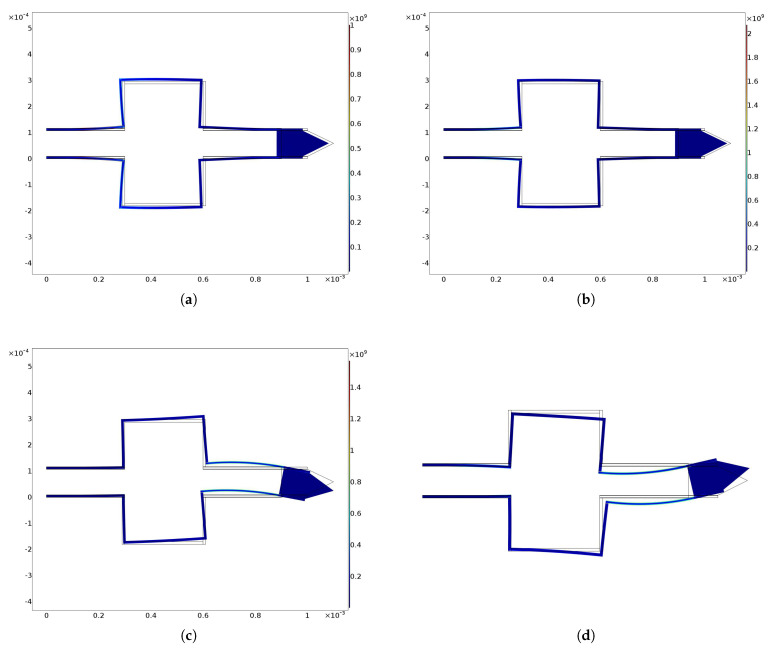
von Mises stress for (**a**) circular, (**b**) elliptical, (**c**) spiral and (**d**) cycloidal trajectories. All graphs were obtained by means of FEM simulations. Stress scale is in GPa.

**Figure 8:**
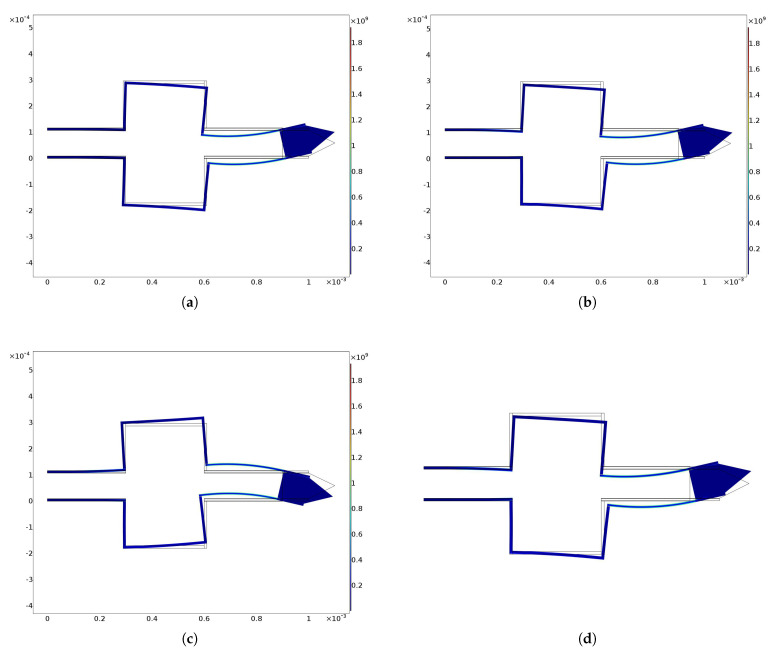
von Mises stress for (**a**) Lissajous 1, (**b**) Lissajous 2, (**c**) Lissajous 3 and (**d**) cosinusoidal/linear trajectories. All graphs were obtained by means of FEM simulations. Stress scale is in GPa.

The von Mises stresses related to previous trajectories are shown in Figure 7 and Figure 8. It can be observed that these are always lower than the pre-yielding stress.

**Figure 9:**
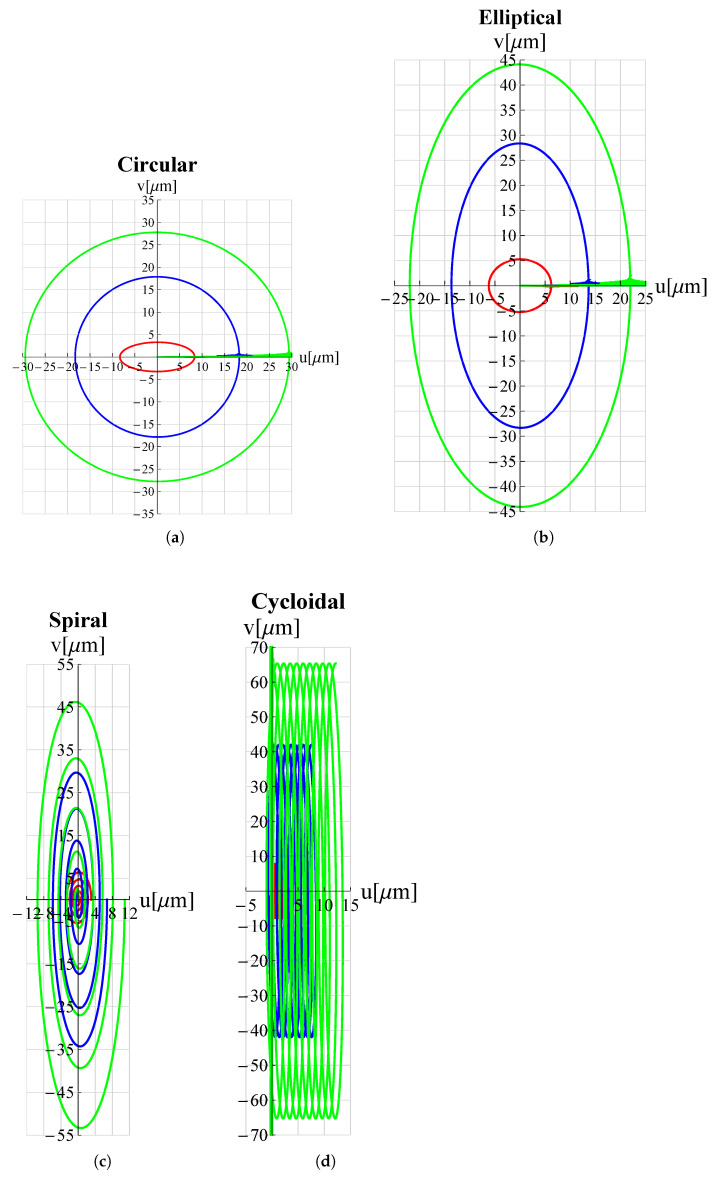
Trajectories for different values of $\overset{\wedge }{h_{c}}$. ——: $\overset{\wedge }{h_{c}}=0.5$; ——: $\overset{\wedge }{h_{c}}=1$; ——: $\overset{\wedge }{h_{c}}=1.5$. (**a**) circular, (**b**) elliptical, (**c**) spiral and (**d**) cycloidal trajectories.

**Figure 10:**
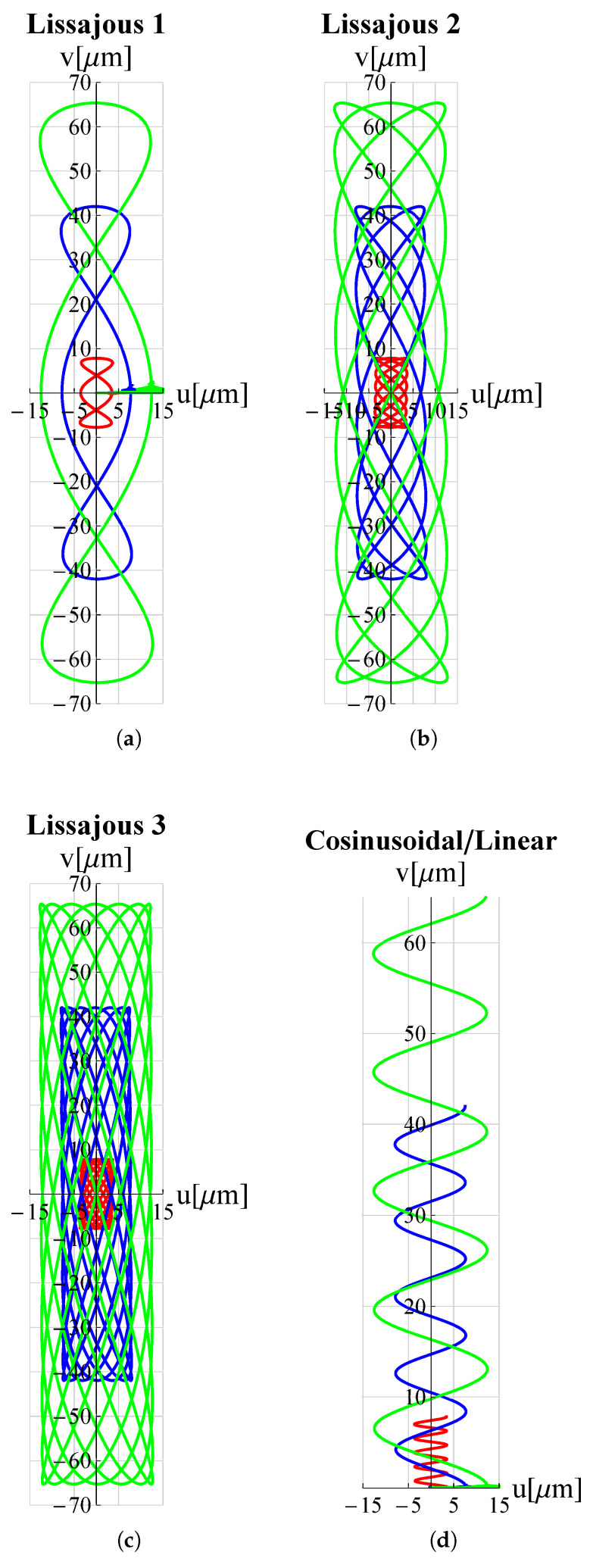
Trajectories for different values of $\overset{\wedge }{h_{c}}$. ——: $\overset{\wedge }{h_{c}}=0.5$; ——: $\overset{\wedge }{h_{c}}=1$; ——: $\overset{\wedge }{h_{c}}=1.5$. (**a**) Lissajous 1, (**b**) Lissajous 2, (**c**) Lissajous 3 and (**d**) cosinusoidal/linear trajectories.

**Figure 11:**
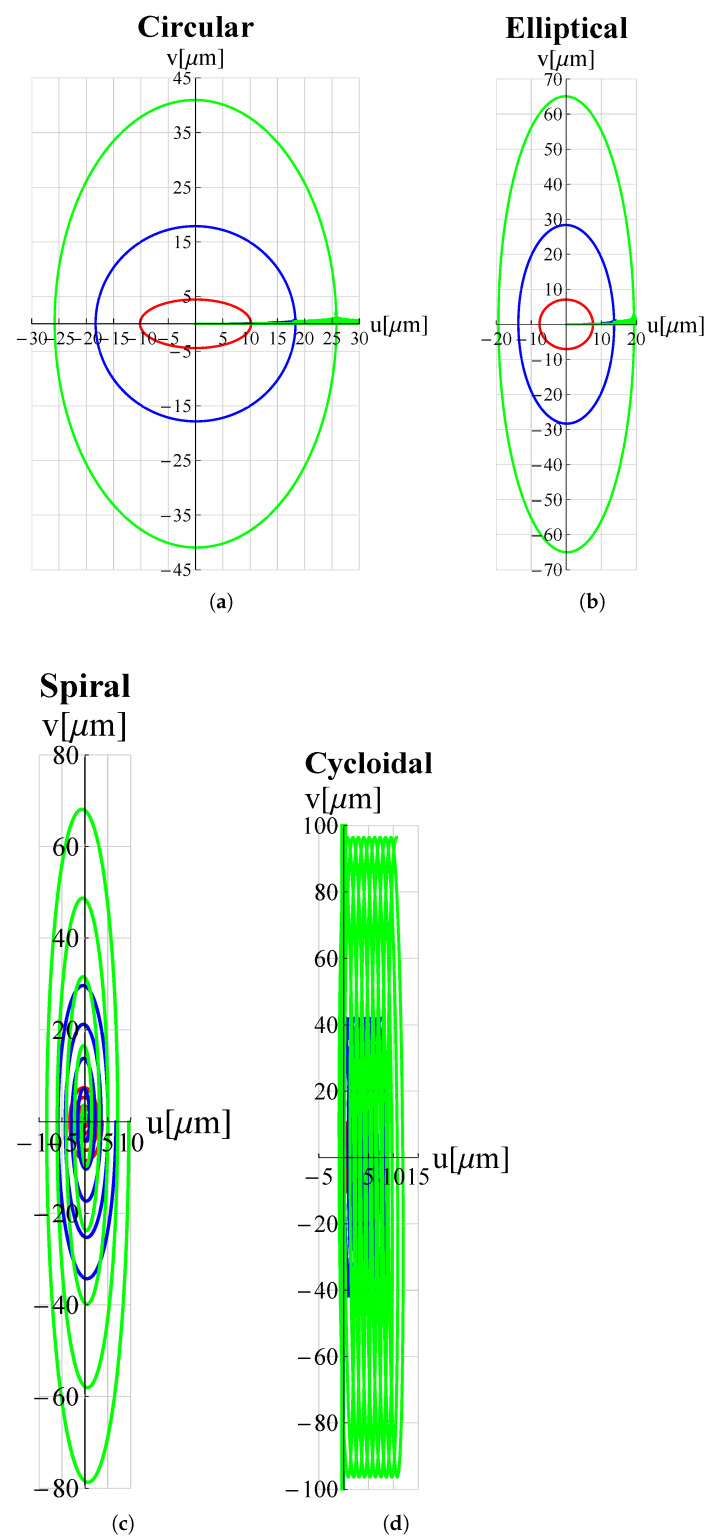
Trajectories for different values of $\overset{\wedge }{L_{1}}$. ——: $\overset{\wedge }{L_{1}}=0.5$; ——: $\overset{\wedge }{L_{1}}=1$; ——: $\overset{\wedge }{L_{1}}=1.5$. (**a**) circular, (**b**) elliptical, (**c**) spiral and (**d**) cycloidal trajectories.

**Figure 12:**
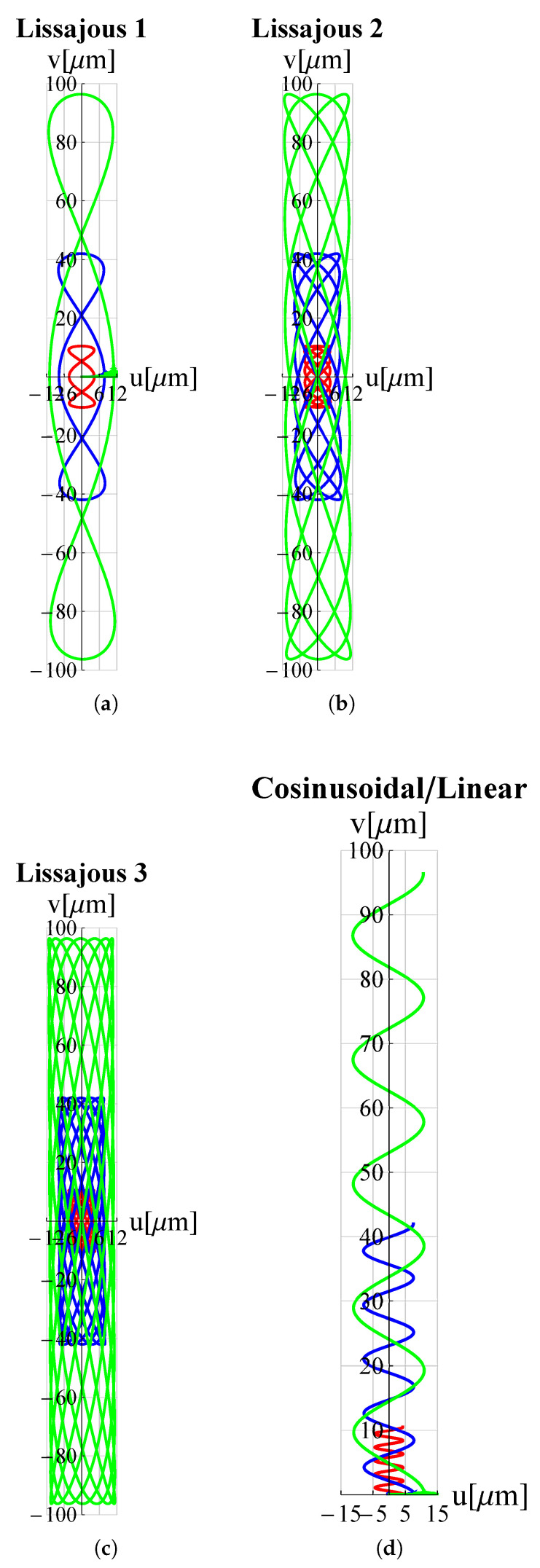
Trajectories for different values of $\overset{\wedge }{L_{1}}$. ——: $\overset{\wedge }{L_{1}}=0.5$; ——: $\overset{\wedge }{L_{1}}=1$; ——: $\overset{\wedge }{h_{c}}=1.5$. (**a**) Lissajous 1, (**b**) Lissajous 2, (**c**) Lissajous 3 and (**d**) cosinusoidal/linear trajectories.

**Figure 13:**
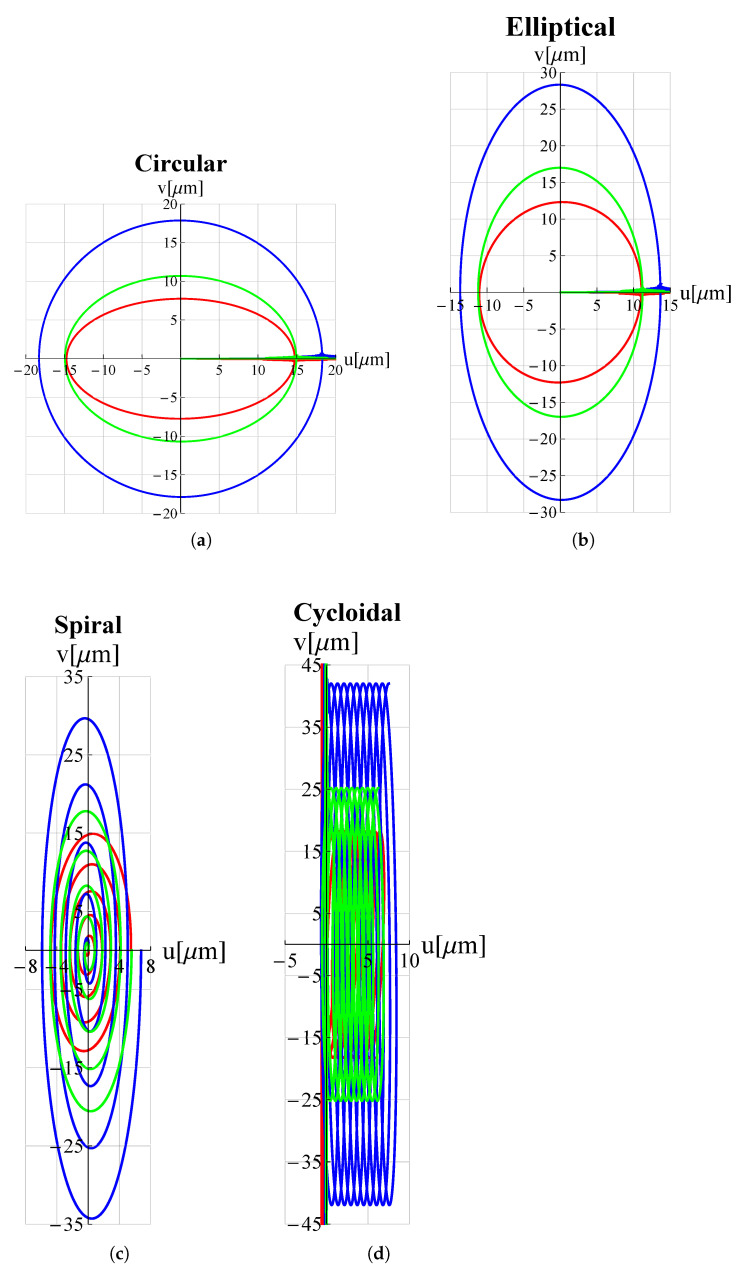
Trajectories for different values of $\overset{\wedge }{h_{1}}$. ——: $\overset{\wedge }{h_{1}}=0.5$; ——: $\overset{\wedge }{h_{1}}=1$; ——: $\overset{\wedge }{h_{1}}=1.5$. (**a**) circular, (**b**) elliptical, (**c**) spiral and (**d**) cycloidal trajectories.

**Figure 14:**
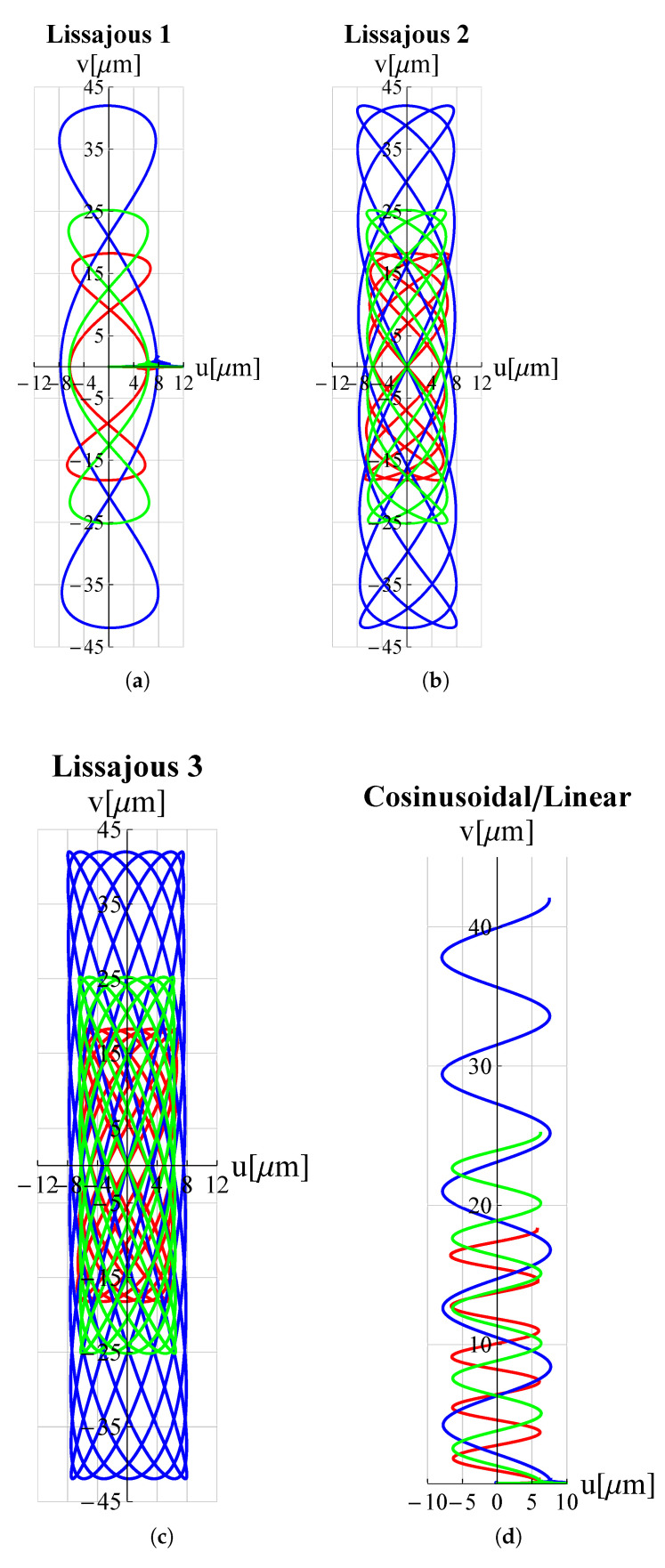
Trajectories for different values of $\overset{\wedge }{h_{1}}$. ——: $\overset{\wedge }{h_{1}}=0.5$; ——: $\overset{\wedge }{h_{1}}=1$; ——: $\overset{\wedge }{h_{1}}=1.5$. (**a**) Lissajous 1, (**b**) Lissajous 2, (**c**) Lissajous 3 and (**d**) cosinusoidal/linear trajectories.

In order to examine the influence of the most significant geometrical parameters on the amplitude of the working range, analyses were performed for different values of $L_{1}$, $h_{c}$ and $h_{1}$. The results are shown in Figure 9, Figure 10, Figure 11, Figure 12, Figure 13 and Figure 14 where $\overset{\wedge }{h_{c}}$, $\overset{\wedge }{L_{1}}$, $\overset{\wedge }{h_{1}}$, $\overset{\wedge }{A_{x}}$ and $\overset{\wedge }{A_{y}},$ indicate the values of $h_{c}$, $L_{1}$, $h_{1}$ and of the working range, along *x* and *y* axes, normalized with respect to the values given in Table 1. It can be observed that as $h_{c}$ and $L_{1}$ increase the working amplitudes, both along *x* and *y*, increase while the trend with $h_{1}$ is different, in fact it can be seen that in this case the maximum values are obtained, both along x and y, for $h_{1}=1$.

**Figure 15:**
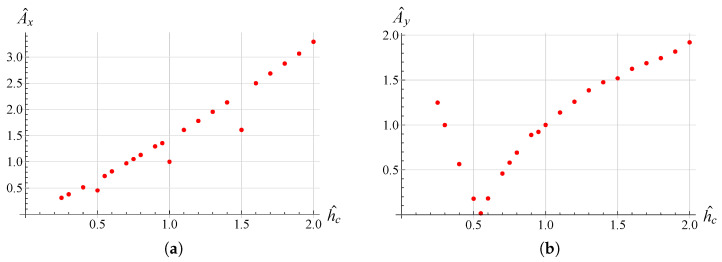
$\overset{\wedge }{A_{x}}$ vs $\overset{\wedge }{h_{c}}$ (**a**), $\overset{\wedge }{A_{y}}$ vs $\overset{\wedge }{h_{c}}$ (**b**) variation for the circular trajectory.

**Figure 16:**
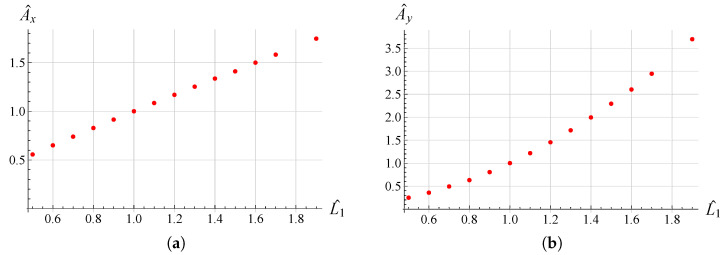
$\overset{\wedge }{A_{x}}$ vs $\overset{\wedge }{L_{1}}$ (**a**), $\overset{\wedge }{A_{y}}$ vs $\overset{\wedge }{L_{1}}$ (**b**) variation for the circular trajectory.

**Figure 17:**
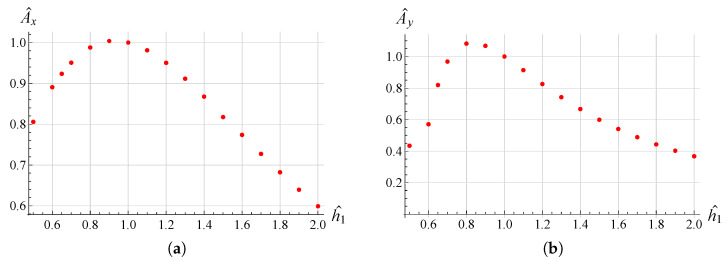
$\overset{\wedge }{A_{x}}$ vs $\overset{\wedge }{h_{1}}$ (**a**), $\overset{\wedge }{A_{y}}$ vs $\overset{\wedge }{h_{1}}$ (**b**) variation for the circular trajectory.

In order to evaluate these behaviours in more detail, a reference trajectory (circular) was chosen, and numerical simulations were performed at multiple points for all three parameters. The results are shown in Figure 15, Figure 16 and Figure 17. It can be observed that there is an established tendency for the working range, on both axes, to increase as $L_{1}$ increases, while, as $h_{c}$ increases, the working amplitude along *x* is increasing with a linear type trend, but along the *y*-axis there is a minimum near $\overset{\wedge }{h_{c}}=0.55$. The results obtained with $h_{1}$ show a different trend, in fact the presence of a maximum value near $\overset{\wedge }{h_{1}}=0.9$ for $A_{x}$ and $\overset{\wedge }{h_{1}}=0.8$ for Ay can be clearly identified.

The device described in this investigation has been analyzed in order to assess its functional characteristics by assuming that the fabrication stage does not affect any of its geometric properties. At the macro scale, any technological process can be used, which may include classical machining, additive manufacturing and also assembling stages. In this case, the device can be still used as a nanomanipulator because, although its maximum size could reach a few tens of cm, its tip could be guided very precisely by regulating the amplitude of the applied voltage functions. At the micro or nano scale the device could be classified as a real micro or nano robot, since its size is reduced to the micro on nano scale (the tip workspace size has the same order of magnitude of the device). The future developments will concern the identification of an appropriate technological process to construct a first prototype of the device, especially at the mico or nano scale, for example, by using Silicon and Aluminum Nitride (AlN) where two symmetric AlN- -Si- -AlN sandwiches could be adopted by taking inspiration by other AlN applications in MEMS [49].

## 4. Conclusions

In this work, a new micro piezo device is proposed. The device is capable of generating any plane trajectory represented by a parametric curve. It is achieved by the action of piezoelectric plates, suitably powered, on a silicon structure, taking advantage of the symmetry, and antisymmetry, of such actions. The future developments include the construction of an initial prototype to carry out initial testing. A wireless distribution system for applications on rotating micro structures is also being studied [50].

## Figures and Tables

**Table 1:** Piezoelectric-based microdevice geometric specifications.

Label	Value (μm)	Label	Value (μm)
$L_{1}$	300	$t_{c}$	100
$L_{2}$	100	$t_{a}$	1
$h_{1}$	7.5	$h_{c}$	180
$t_{f}$	10	$w_{t}$	$L_{1}/3$
$L_{p}$	Lg		
out-of-plane thickness			7.5

**Table 2:** Materials properties.

Property	Silicon	PZT-5A	Unit
Density	2329	7750	kg/m${}^{3}$
Poisson’s ratio	0.28	–	–
Young’s modulus	170	65	GPa
$d_{31}=d_{32}$	–	−1.71	$10^{-10}$ C/N
$d_{33}$	–	3.74	$10^{-10}$ C/N
$d_{51}=d_{42}$	–	5.84	$10^{-10}$ C/N

**Table 3:** Voltage functions $V_{x}\left(t\right)$ and $V_{y}\left(t\right)$ required to generate the desired pathways.

Trajectory			Voltage Functions	
Label	$f_{x}\left(t\right)$	$f_{y}\left(t\right)$	$V_{x}\left(t\right)$	$V_{y}\left(t\right)$
Circular	acos(t)	asin(t)	40cos(t)	17sin(t)
Elliptical	acos(t)	bsin(t)	30cos(t)	27sin(t)
Spiral	$a\left(e^{\frac{t}{10}}-1\right)\cos\left(5t\right)$	$b\left(e^{\frac{t}{10}}-1\right)\sin\left(5t\right)$	$17\left(e^{\frac{t}{10}}-1\right)\cos\left(5t\right)$	$40\left(e^{\frac{t}{10}}-1\right)\sin\left(5t\right)$
Cycloidal	$\frac{a}{2\pi }\left(t+\sin\left(10t\right)\right)$	bcos(10t)	$\frac{17}{2\pi }\left(t+\sin\left(10t\right)\right)$	40cos(10t)
Lissajous 1	$a\sin(3t+\frac{\pi }{2})$	bsin(t)	$17\sin(3t+\frac{\pi }{2})$	40sin(t)
Lissajous 2	asin(8t)	bsin(3t)	17sin(8t)	40sin(3t)
Lissajous 3	asin(10t)	bsin(7t)	17sin(10t)	40sin(7t)
Cosinusoidal/Linear	acos(5t)	$b\frac{t}{2\pi }$	17cos(5t)	$40\frac{t}{2\pi }$

## Data Availability

Not applicable.
